# Laparoscopic treatment in a patient with median arcuate ligament syndrome identified at the onset of superior mesenteric artery dissection: a case report

**DOI:** 10.1186/s40792-019-0758-7

**Published:** 2019-12-11

**Authors:** Haruhiko Okada, Kazuhisa Ehara, Hisashi Ro, Masaki Yamada, Tetsuya Saito, Naoki Negami, Yasunori Ishido, Masahiko Sato

**Affiliations:** 1 Department of Surgery, Saiseikai Kawaguchi General Hospital, 5-11-5 Nisikawaguchi, Kawaguchi, Saitama, 332-8558 Japan; 2 Division of Gastroenterological Surgery, Saitama Cancer Center, 780 Komuro, Ina, Kita-adachi gun, Saitama, Saitama Prefecture 362-0806 Japan; 3 Department of Coloproctological Surgery, Juntendo University, Tokyo, Japan

**Keywords:** Median arcuate ligament syndrome, Superior mesenteric artery dissection, Laparoscopy

## Abstract

**Background:**

Median arcuate ligament syndrome (MALS) is a rare clinical entity caused mainly by extrinsic compression of the celiac axis by the median arcuate ligament (MAL). Severe celiac artery stenosis can lead to the development of collateral circulation, aneurysms, and, rarely, superior mesenteric artery (SMA) dissection. The treatment of MALS involves the surgical release of the MAL. However, a standard procedure with the use of laparoscopy has not been established, and intraoperative complications can lead to severe vascular injury.

**Case presentation:**

The patient was a 43-year-old man with MALS identified at the onset of SMA dissection. After treatment for the SMA dissection, he underwent laparoscopic MAL release. Using the technique of laparoscopic gastrectomy within the surgical field, we performed laparoscopic MAL release and ganglionectomy safely with a good view. Immediate symptomatic improvement was acquired, and no recurrence was observed at the 20-month follow-up.

**Conclusion:**

We reported a rare case of MALS and SMA dissection. A horizontal 3D laparoscopic approach of the celiac axis allows for safe, meticulous, and radical MAL release and ganglionectomy.

## Background

Median arcuate ligament syndrome (MALS) is a rare clinical entity characterized by chronic intermittent abdominal pain triggered by meals, nausea, and weight loss [1].

Extrinsic compression of the celiac artery by fibers of the median arcuate ligament (MAL) causes this syndrome, but the etiology of MALS is largely debated [2, 3].

Another important aspect of MALS is the risk of developing collateral circulation that leads to aneurysm rupture [4]. Superior mesenteric artery (SMA) dissection has rarely been reported [5]. Definitive treatment for MALS involves surgical release of the MAL, including celiac ganglion fibers. Recently, a laparoscopic approach has been used by surgeons due to its minimally invasive manner, but a standard procedure for laparoscopic MAL release has not been established because of the rarity of MALS and the rate of complications or relapse [6–8].

We present a case of laparoscopic MAL treatment in a patient with MALS identified at the onset of superior mesenteric artery (SMA) dissection using the technique of gastrectomy within the surgical field.

## Case presentation

The patient was a 43-year-old man who visited the emergency department with severe abdominal pain he had never had before 3 h after acute onset. His medical history included hypertension and recurrent episodes of severe postprandial or preprandial epigastric pain for a few years. The results of esophagogastroduodenoscopy, abdominal ultrasound, and CT were all unremarkable.

**Fig. 1:**
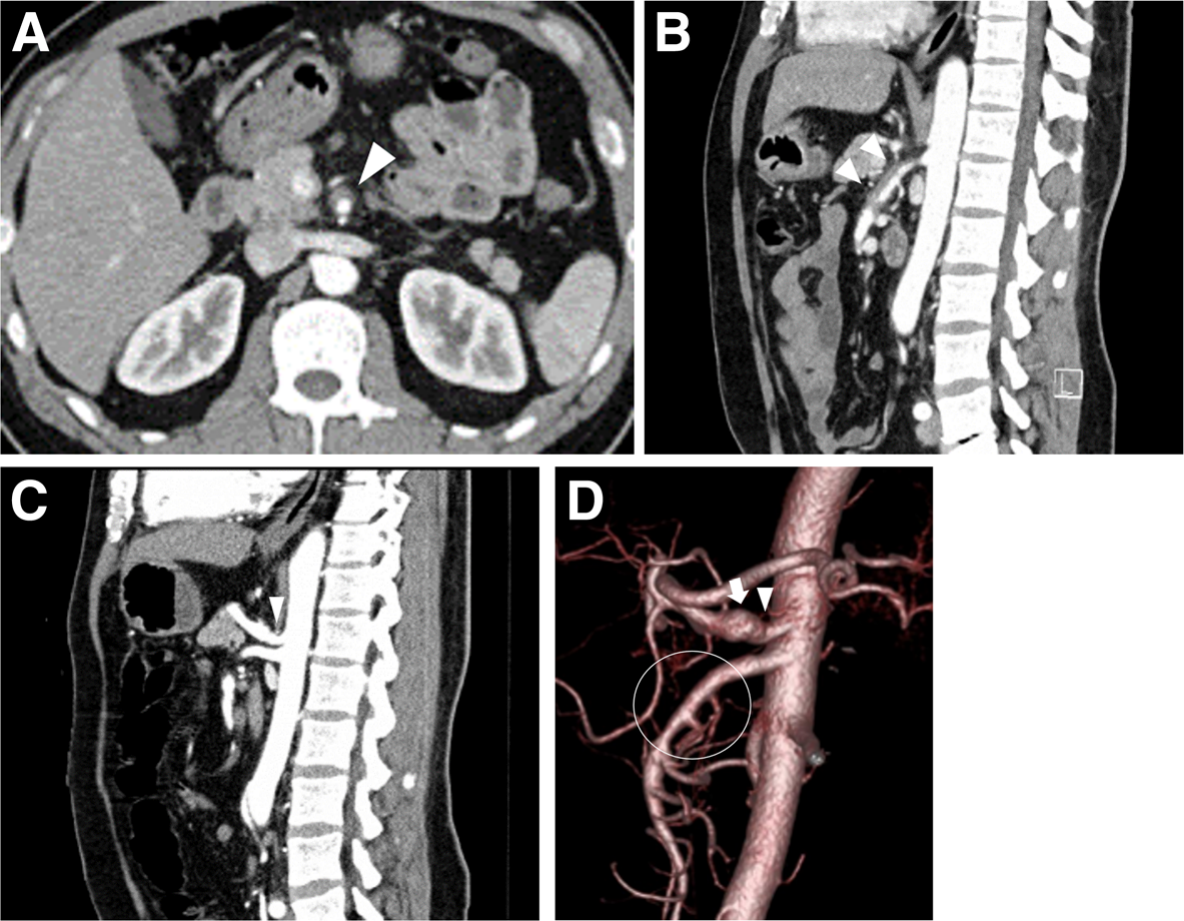
**a**, **b** Superior mesenteric artery dissection with the thrombosed false lumen is shown by arrow heads on an axial view (**a**) and a sagittal view (**b**) of contrast-enhanced CT. **c** Sagittal view of enhanced CT showing extrinsic compression of the root of celiac axis by the MAL (arrowhead). **d** 3D reconstruction of abdominal aortic angiotomography showing severe stenosis of the proximal segment of the celiac axis caused by extrinsic compression of the median arcuate ligament (arrow head) and poststenotic dilatation (arrow), and narrow true lumen of superior mesenteric artery(circle) from isolated dissection with a thrombosed false lumen

Abdominal examination revealed epigastric tenderness. No other abdominal findings, such as rebound tenderness or muscular defense, were observed. Contrast-enhanced abdominal CT revealed isolated SMA dissection with a thrombosed false lumen and celiac artery aneurysm (Fig. 1a, b). Two-day conservative treatment with fasting and blood pressure control showed no ischemic sign in the small intestine was observed. A sagittal view of the CT angiography showed extrinsic compression of the root of the celiac axis by the MAL (Fig. 1c), and 3D CT angiography showed proximal celiac axis stenosis and poststenotic dilatation (Fig. 1d). Further conservative treatment relieved his symptoms, and a 3-month follow-up CT showed disappearance of the SMA dissection. After treatment for the SMA dissection, intermittent epigastric pain persisted. Point tenderness with an echoic probe showed that the root of the celiac axis was associated with the most pain and that the symptom was thought to be due to MALS. He opted for laparoscopic MAL release 10 months after treatment for the SMA dissection.

**Fig. 2:**
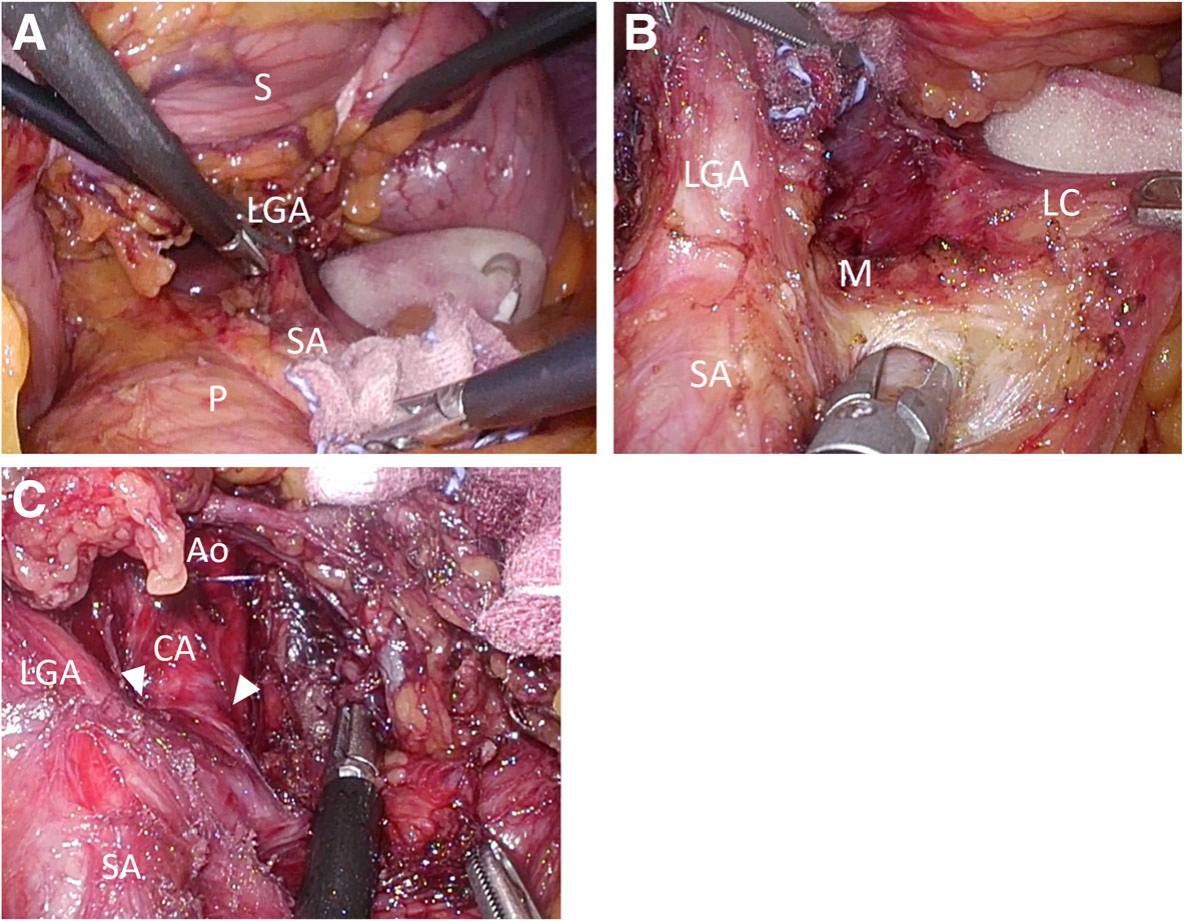
**a** Intraoperative image showing horizontal approach with retracting the stomach ventrally. S, stomach; P, pancreas; LGA, left gastric artery; SA, splenic artery. **b** Intraoperative image showing the fibers of the MAL. M, MAL; LGA, left gastric artery; SA, splenic artery; LC, left crus. **c** Intraoperative image showing the origin of the celiac artery freed from the MAL (arrow head). Ao, arota; CA, celiac artery; LGA, left gastric artery; SA, splenic artery

The procedure was performed under general anesthesia. The patient was placed in a reverse Trendelenburg position with his legs spread apart. The first port of the videoscope was inserted via an open technique at the umbilicus. After the pneumoperitoneum was established, four operating ports were inserted. The left segment of the liver was retracted using a Nathanson Hook Liver Retractor (Yufu ITONAGA CO., LTD. Tokyo Japan). After division of the gastrohepatic ligament, the right crus of the diaphragm was identified, and the peritoneal line anterior to the right crus was opened. Thereafter, the greater omentum was divided, and the left gastric artery was exposed on the suprapancreatic surface. The stomach was raised and retracted ventrally, with taping on both sides of the left gastric artery. One suture was made for fixation of the stomach to the peritoneum. These procedures freed the surgeons’ and assistants’ hands and allowed for caudal-to-ventral MAL division with a good surgical view (Fig. 2a) [9].

After skeletonization of the vessels (common hepatic artery, splenic artery, and left gastric artery) and diaphragmatic crura, all tissues overlying the aorta, commonly referred to as the MAL (Fig. 2b), were divided in a caudal to ventral direction. The MAL was divided to the start of the celiac artery, ensuring that no surrounding musculo-fibrous or periganglionic tissue remained (Fig. 2c). The operative time was 152 min, and the amount of blood loss was 22 ml. The patient was discharged on postoperative day 2 but was readmitted on postoperative day 7 because of gastroparesis, which improved with conservative treatment and fasting.

**Fig. 3:**
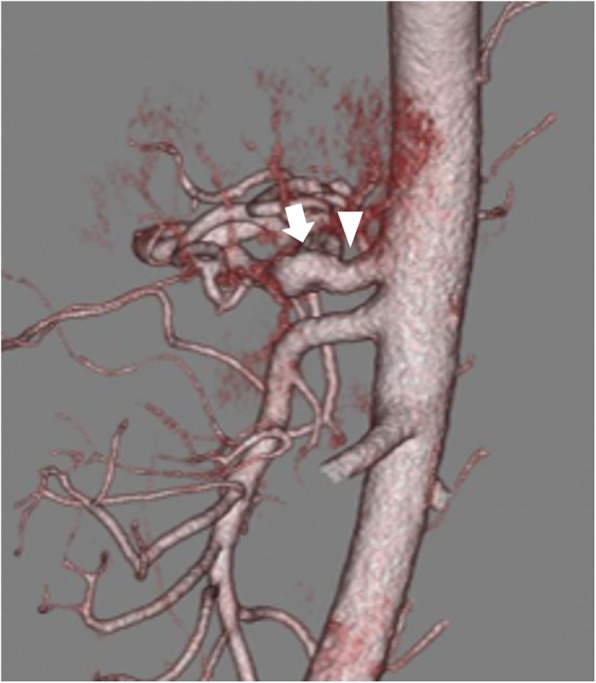
Follow-up (6 months) 3D aortic reconstruction showing a normal caliber of the celiac artery, without evidence of stenosis (arrow head) or poststenotic dilatation (arrow)

After surgery, immediate symptomatic improvement was acquired. Enhanced CT performed 6 months after the surgery revealed no residual celiac axis stenosis or poststenotic dilatation (Fig. 3). At the 20-month follow-up, the patient showed no recurrence of symptoms.

## Discussion

MALS, also known as celiac artery compression syndrome or Dunbar’s syndrome, was first described by Harjola (1964), followed by Dunbar (1965) [1]. Extrinsic compression of the celiac axis by the MAL in patients with an abnormally low insertion of the diaphragm is thought to be the main cause of MALS. Postprandial epigastric pain, nausea or vomiting, and weight loss are commonly observed in patients with MALS. MALS is a diagnosis of exclusion, and it should be considered a possibility in young female patients with abdominal pain of unclear etiology [2, 3]. However, the clinical presentation varies, and the pathophysiology of MALS remains largely unknown. MALS is considered a complex condition with a multifactorial etiology and mechanism of pain. The most accepted theory is that increased blood demand due to a compressed celiac artery leads to foregut ischemia, and another theory is that midgut ischemia causes pain through steel syndrome, wherein blood from the SMA area is diverted through the collateral circulation to compensate for inefficient blood flow from the celiac artery. A third theory is that direct chronic irritation of the celiac plexus or indirect overstimulation of the celiac plexus through celiac ganglion compression by the MAL causes subsequent visceral vasoconstriction and ischemia [2]. Currently, complete celiac trunk decompression is recommended and only simple MAL release is not enough, additional neurolysis and wide excision of the celiac plexus is necessary [7]. These procedures have been reported to not only better inhibit reformation of a compression but also the result in a decrease of the pain associated with MALS [7]. However, detecting celiac trunk stenosis with routine axial and coronal reconstructed CT is difficult, and sagittal reconstruction is necessary [4]. The incidence of MALS is 0.4%, and the majority of people with extrinsic compression of the celiac axis by the MAL on computed tomography (CT) remain asymptomatic [4].

Significant stenosis of the celiac artery leads to increased blood flow into the SMA, which causes the formation of collateral circulation that maintains blood flow to the organs and subsequent aneurysm formation. Once the formation of splanchnic artery aneurysms is developed, prophylactic embolization may be considered because of the risk of rupture [4]. Poststenotic dilation of the celiac trunk is related to hemodynamically significant stenosis. Our patient also had poststenotic dilation of the celiac trunk and fortunately no collateral circulations or aneurysms, and he was accidentally diagnosed with MALS at the onset of SMA dissection, which may have occurred from increased blood flow. There are only three cases documented for the MALS and SMA dissection combination [5].

Since the introduction of laparoscopic MAL release in 2000 [6], minimally invasive approaches have been selected by surgeons. Nevertheless, a standard procedure for MAL release has not been established because of the rarity of MALS. Most surgeons prefer to approach the MAL only through the gastrohepatic ligament with retraction of the stomach caudally [2, 6, 7]. The problems with the laparoscopic approach, however, are the restriction of the instrument’s maneuverability and the presence of a confined space. The complication rate of laparoscopic MAL release is relatively high (7.3%) compared with that of robotic MAL release (5.3%), and the most common intraoperative complication is vascular injury. The rate of symptom recurrence is reported to be 9% [7, 8]. To overcome this difficulty, we selected the retraction of the stomach ventrally which freed the surgeons’ and assistants’ hands and the horizontal surgical approach for the celiac artery through the suprapancreas with the use of 3D laparoscopy. Exposure and skeletonization of the vessels with these procedures were relatively similar to that in laparoscopic gastrectomy via a medial approach [9, 10]. The meticulous and straightforward procedure with no organ obstruction allows for safe and definite MAL release and ganglionectomy, which lead to the resolution of long-term MALS symptoms.

## Conclusion

In summary, we described a rare case of MALS identified at the onset of SMA dissection. Using the technique of laparoscopic gastrectomy within the surgical field, we performed safe, meticulous, and radical MAL release and ganglionectomy.

## Data Availability

Not applicable

## Abbreviations

CT: Computed tomography
MAL: Median arcuate ligament
MALS: Median arcuate ligament syndrome
SMA: Superior mesenteric artery
